# Pancreatic Necrosis Infection as a Determinant of Multiple Organ Failure and Mortality in Acute Pancreatitis

**DOI:** 10.3390/pathogens12030428

**Published:** 2023-03-08

**Authors:** Igor A. Kryvoruchko, Valeriy V. Boyko, Massimo Sartelli, Yulia V. Ivanova, Denys O. Yevtushenko, Andrij S. Honcharov

**Affiliations:** 1 Department of Surgery No.2, Kharkiv National Medical University, Nezalezhnosti Avenue, 61022 Kharkiv, Ukraine; ashoncharov.po22@knmu.edu.ua; 2 Institute General and Emergency Surgery Named after V.T. Zaitcev of the National Academy of Medical Sciences of Ukraine, Balakireva Entry, 61103 Kharkiv, Ukraine; igusurg@ukr.net; 3 Department of Surgery No.1, Kharkiv National Medical University, Balakireva Entry, 61103 Kharkiv, Ukraine; dr.ivanova23@gmail.com (Y.V.I.); dr.yevtushenko@ukr.net (D.O.Y.); 4 Department of Surgery, Macerata Hospital, Santa Lucia Street, 62100 Macerata, Italy; massimosartelli@gmail.com

**Keywords:** acute necrotizing pancreatitis, pancreatic infection, treatment, bacterial strains, antibiotics, multiple organ failure, mortality

## Abstract

Several recommendations and data on the treatment of acute necrotizing pancreatitis (ANP) are conflicting and different surgical approaches continue to exist. We conducted a study on 148 patients with ANP, who were divided into two groups: the main group (*n* = 95) when the tactics of the step-up approach were applied with the principles of the concept of Enhanced Recovery After Surgery (ERAS) in order to determine this approach on effectiveness in reducing complications and 30-day mortality (2017–2022); the comparison group (*n* = 53) when the same tactic of the treatment was used without ERAS principles (2015–2016). Treatment time for the main group in the intensive care unit was minimized (***p* ≤ 0.004**); it has been shown to reduce the frequency of complications in these patients (***p* < 0.001**) requiring conservative or surgical treatment without general anaesthesia (Clavien-Dindo I-IIIa); no statistically significant differences were observed for the total incidence of Clavien-Dindo IIIb-IVb complications (*p* > 0.05); the median duration of treatment for patients in the primary group was 23 days, and in the reference group—34 days (***p* ≤ 0.003**). Pancreatic infections have been observed in 92 (62.2%) patients and gram-negative bacteria predominated in the overall pathogen structure with 222 (70.7%) strains. The only evidence of multiple organ failure before (**AUC = 0.814**) and after surgery (**AUC = 0.931**) was found to be predictive of mortality. Antibiotic sensitivity of all isolated bacteria better understood local epidemiology and identified the most effective antibiotics when treating patients.

## 1. Introduction

Acute pancreatitis (AP) is one of the most urgent and complicated problems in emergency abdominal surgery and is the most common pancreatic disease in the world. The epidemiological estimates presented in the study [1] indicate that incidence is on the rise globally. According to data, 33–74 cases per 100,000 people/year were found in various countries of the world and 1–60 deaths per 100,000 people/year in AP, and according to the conducted studies, the overall incidence of AP has increased by 3.07% in a year for the last 56 years, contributing to an increase in the burden on health care systems [2]. Although the overall mortality in AP in leading pancreatological centers does not exceed 5–6%, in the event of acute necrotizing pancreatitis (ANP), purulent-septic complications and sepsis, it is 20–45.4% and does not change, despite successes in improving diagnostics, intensive therapy and introduction of new methods of surgical treatment [3]. In recent years, the frequency of ANP has increased (up to 15–30%), as well as infected forms of ANP (up to 40–80%), and the severity of the disease and mortality begins with the occurrence of sepsis and multiple organ failure (MOF), so the presence of infected pancreatic necrosis is an absolute indication for surgery [4].

In different periods, the treatment of AP has changed from being strictly conservative to the use of various methods of surgical treatment [5,6]. Modern surgical tactics for the treatment of local complications of ANP are based on the widespread implementation of minimally invasive procedures (MIP) [6,7], and the world community of pancreatologists has initiated the introduction of a step-up approach tactic to the surgical clinic, and includes percutaneous drainage, transluminal endoscopic necrosectomy through the stomach or duodenum, laparoscopic necrosectomy, retroperitoneal surgical drainage, etc. [8], as independent surgical methods of treatment in the presence of pancreatic and peripancreatic accumulation of fluid formations and pseudocysts, or as a sequential staged approach (step-up approach) of preparation for necrosectomy in the case of infection, which is consistent with the principles of international recommendations [9,10]. The application of the step-up approach tactic in clinical practice is closely intertwined with the implementation of the concept of multimodal rehabilitation of surgical patients through the implementation of the protocols for Enhanced Recovery After Surgery (ERAS) or fast-track surgery. This approach can provide tools to improve outcomes and decrease the cost of ANP treatment without compromising its quality. The ERAS concept provides a set of peri- and postoperative measures to reduce hospital and rehabilitation time after surgery [11]. Since patients with AP are the category of patients who most often need long-term and costly inpatient treatment, attempts to implement the concept of ERAS during their treatment are relevant and cost-effective.

The primary aim of the study was to determine the effectiveness of ERAS principles in reducing complications and 30-day mortality during the ANP treatment stages.

The secondary aim of the study was to evaluate the microbiological characteristics of ANP and the antibiotic sensitivity of all isolated bacteria, to better understand the local epidemiology and define the most effective antibiotics.

## 2. Materials and Methods

### 2.1. Study Design and Patient Selection

A two-center case-control study was conducted on 148 patients with ANP at Kharkiv National Medical University from 1 January 2015 to 30 October 2022. All subjects gave their informed consent for inclusion before they participated in the study. The study was conducted following the Declaration of Helsinki, and the protocol was approved by the Ethics Committee of Kharkiv National Medical University (Protocol No. 6, 11 November 2022). The project identification code is 0116u00499. All patients were divided into two groups: the main group (*n* = 95) and the comparison group (*n* = 53). In the main group, the tactics of the step-up approach were applied and the principles of the ERAS concept (2017–2022) were implemented. In the comparison group (2015–2016), the ERAS principles were not implemented. The classification of the AP was used according to the recommendations of the International Consensus [12]. The inclusion criteria were: (1) proven ANP in both phases (early (<1 week) and late (>1 week) after onset of the disease, (2) using any MIP in patients due to their treatment, and (3) 18 ≤ age ≤ 70 years. The exclusion criteria were: (1) patients with postoperative AP; and (2) those who refused to participate in the study.

### 2.2. Data Collection

Medical records, including symptoms and signs, laboratory tests and imaging studies, were reviewed. Written informed consent was obtained from each patient. The primary endpoint was recovery without surgical intervention. Secondary endpoints were developments in MOF and 30-day mortality. The diagnosis of AP was established based on the analysis of the anamnesis data, and the results of clinical, laboratory, and instrumental (ultrasound, CT scan with intravenous contrast) research methods. The severity of ANP was assessed according to the recommendations of the AP classification revision group (2012) [12].

### 2.3. Management of ANP

In both patient groups, we adhered to international guidelines for the treatment of AP adapted to our local resources and procedures [9]. Controversies regarding the management of ANP have been described [13]. Fluid therapy, nutritional supplements—we mainly used delayed oral feeding due to 72 h after hospital admission in patients with moderate ANP and insertion of a nasojejunal tube (within 24 h after admission) in patients with severe ANP using a low-fat diet. Several of hemodynamically stable patients with intolerance to nasojejunal feeding, received total parenteral nutrition. All patients were also given systemic support, analgesia, and antibiotics as needed for (1) a confirmed pancreatic infection, (2) if an infection was suspected due to worsening patient conditions during intensive treatment in the intensive care unit, and (3) during the use of percutaneous drainage with a collection of aseptic fluid. Indications for performing minimally invasive interventions were considered to be proven infection of pathological foci of ANP (the presence of gas according to CT data) and after a positive microbiological result of a sample collected by aspiration under ultrasound or CT navigation, or the assumption of infection when the clinical course of the disease worsens with the appearance of a new organ failure, or for the duration of organ failure for several weeks with conservative treatment and the appearance of intra-abdominal hypertension. If, despite the use of complex conservative therapy, laparoscopy, endoscopy, percutaneous methods of surgical treatment, and open mini-accesses, the disease progressed or marked reduced phases of the disease with rapid suppuration of tissue in the retroperitoneal space, increased severity of intoxication, or the appearance of surgical complications, proceed to the next stage of treatment, namely, the performance of a wide laparotomy, VAC therapy, and sometimes to the programmed lavage of the abdominal cavity. The step-by-step implementation of the concept of multimodal rehabilitation of patients after minimally invasive surgical interventions included several main stages: the first stage was the minimum length of stay of patients after surgical intervention in the intensive care unit; the second stage was the use of multimodal analgesia to provide adequate analgesia by prolonging epidural anaesthesia on the ThVII-ThVIII levels when using a pump for constant injection of an anaesthetic; the third stage was the activation of the patient, which started from the first day of the postoperative period; the fourth stage was active involvement in the treatment process of the physiotherapeutic service (inhalations with antiseptic solutions, physical therapy, vibromassage, etc.); the fifth stage was the early start of oral intake of clean liquids and enteral nutrition (with the aim of adequate protein and energy supply). The ERAS protocol was modified (Table 1) based on the analysis of the literature data [14,15,16]. Potential barriers to the success of ERAS interventions in patients with acute pancreatitis of varying severity included problems with systematic follow-up of protocol progress as well as the limited resources of this healthcare system in Ukraine. Key elements to consider when implementing successful ERAS protocols for this patient population included: (1) organization of a team consisting of surgeons and resuscitators, other healthcare professionals, and trained nurses; (2) development of interventions based on the systematically assessed high-quality literature, since many patients with ANP need staged treatment, including reoperations, and (3) the introduction of a clear methodology for regularly updating protocols and checking the success or potential limitations of this intervention.

Postoperative complications were assessed according to the Clavien-Dindo classification [17].

### 2.4. Statistical Analysis

Statistical data processing was performed using version 13.3 of STATISTICA. Initially, statistical analysis was performed using descriptive statistics to assess the normality of the distributions of the selected indicators. Continuous data were presented as the median and interquartile range (IQR). The significance of the connections between the crosstalk variables was estimated using criterion χ2. In all cases, the verification of statistical hypotheses was conducted with a confidence level of 95% or more. The capability of MOF as a biological marker to predict mortality was analyzed by the receiver operating characteristic (ROC) curve. The area under the ROC curve (AUC) and the respective confidence interval (CI) was used as a measure of the overall index accuracy by the automated backward stepwise selection of parameters, and the significance of the differences between them was assessed and taken into account in its 95% confidence interval. The prognostic efficacy of the models was assessed by discrimination based on the AUC index. The efficacy of the model was considered limited at AUC > 0.70, good at AUC > 0.80, and excellent at AUC > 0.90.

## 3. Results

### 3.1. Study Characteristics and Patient Demographics

The basic demographic, clinical, and laboratory data are presented in Table 2.

**Figure 1:**
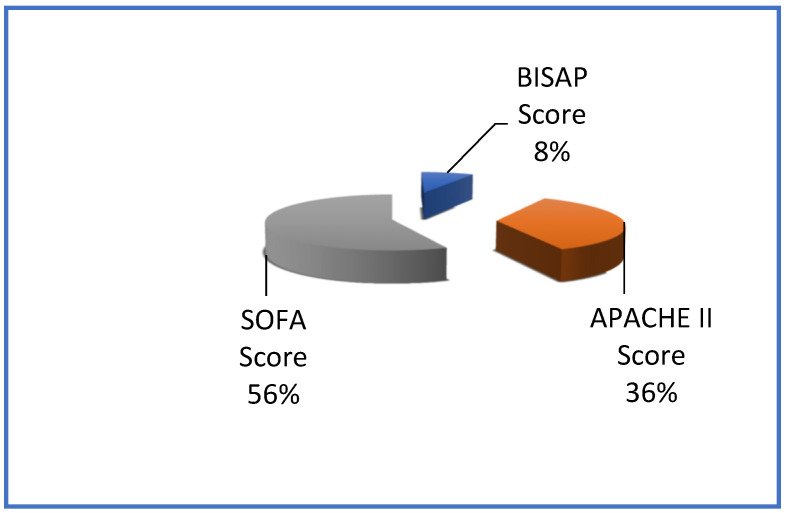
Assessment of severity and predicted mortality of both groups.

Of the patients examined, metabolic ANP was diagnosed in 61.5% of both groups, cholelithiasis represented 35.1% of cases, and other causes accounted for 3.8%. In 60 (40.5%) patients of both groups, a severe form of ANP was diagnosed, which was characterized by persistent organ failure (lasting more than 48 h), predominantly respiratory and cardiovascular, and subtotal and total damage to the pancreas, which in 43 patients was accompanied by damage to the peripancreatic fibre. The overall condition of 88 patients (59.5%) was evaluated as moderate ANP; organ failure was transient and resolved in two days. The projected severity and mortality assessment for both groups is shown in Figure 1.

**Figure 2:**
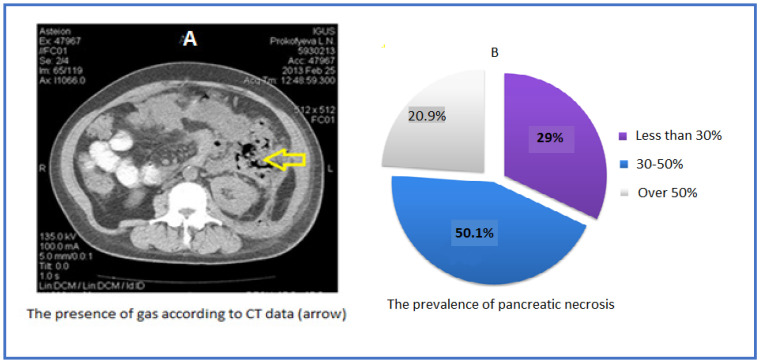
Abdominal CT scan with intravenous contrast (**A**); extension of necrosis in analyzed patients (**B**).

Thirty-eight (25.6%) of 148 patients had sterile ANP, 18 (12.2%) patients had sterile local complications, and secondary pancreatic infection was detected in 92 (62.2%) patients. 59.5% of 148 patients had transient MOF, 40.5% had persistent MOF, 50.1% had 30%–50% pancreatic necrosis on the CECT of the abdomen, whereas 20.9% had over 50% necrotic pancreas (Figure 2).

### 3.2. Surgery Procedures Characteristics

#### Initial Resuscitation and Management of Local Complications

**Figure 3:**
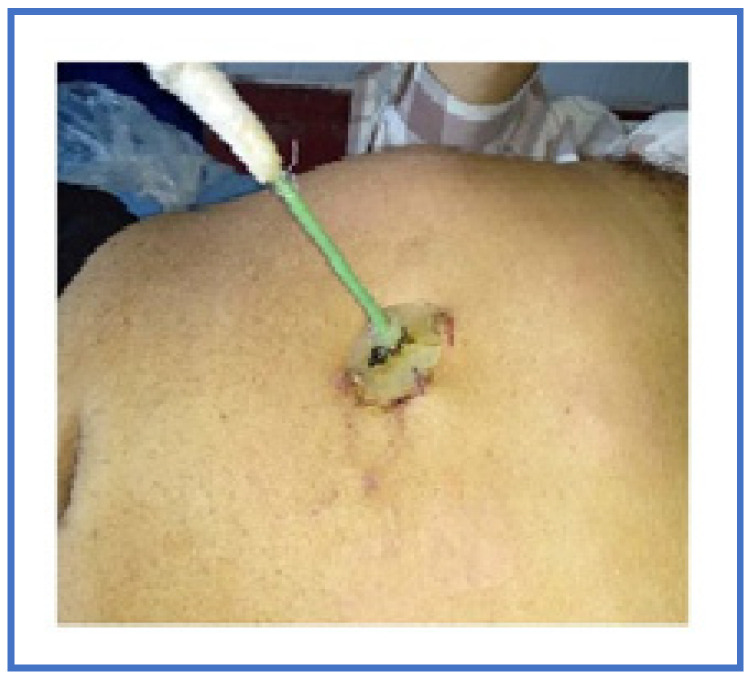
Percutaneous catheter drainage in an infected PS.

**Figure 4:**
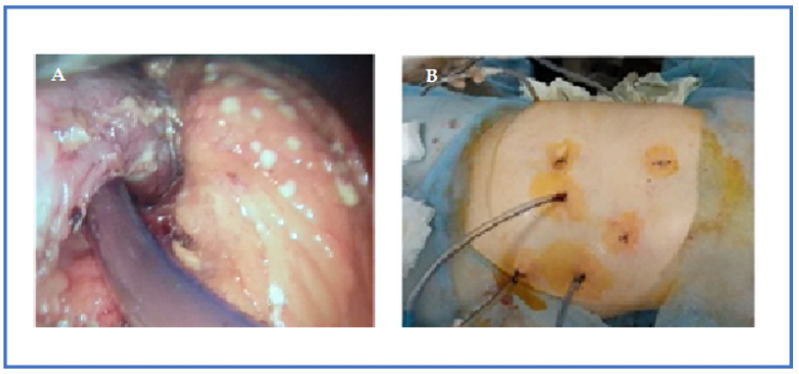
Videolaparoscopy and drainage of multiple infected fluid collections in ANP: endophoto (**A**) and final view of the operation (**B**).

**Figure 5:**
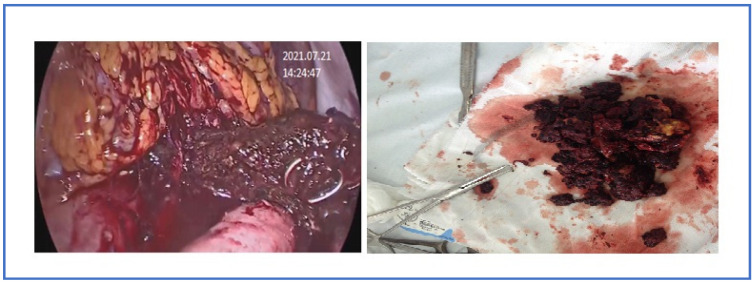
Videolaparoscopy and necrosectomy through the mesocolon in an infected ANP.

A total of 38 (25.6%) of 148 patients with sterile ANP were treated conservatively, 8 (21.1%) of whom were been undergoing endoscopic papillosphincterotomy and choledocholithoextraction due to choledocholithiasis without cholangitis after normalization of clinical and laboratory parameters; 4 (10.5%) were operated on 3–4 weeks after the onset of the disease due to the development of a secondary pancreatic infection. Patients who had pancreatic/peripancreatic collection(s) with persistent infection, infected necrosis, persistent MOF, or clinical deterioration (development of MOF, fever, leukocytosis, or locoregional pressure effects even with sterile collection) were considered for image-guided percutaneous catheter drainage (PCD) and/or laparoscopic necrosectomy/drainage (LD), and the presence of infected necrosis was not the only indication for it (Figure 3 and Figure 4). PCD or LD insertion was considered the first step in the therapy to decrease the toxic load from the disease if the collection were over 6 cm for performing a necrosectomy in parts of the patients for 3–4 weeks after the onset of the disease (Figure 5).

**Figure 6:**
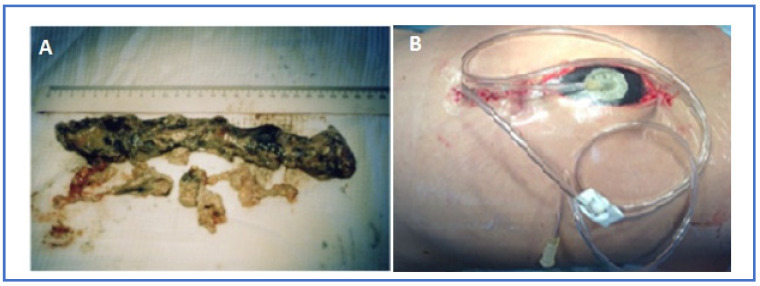
Necrosectomy (**A**) and decompressive VAC—laparostomy (**B**) in a patient with infected ANP.

Of 148 patients, 10.1% (*n* = 15) developed WON with fluid accumulation and therefore required PCD (*n* = 8, the main group vs the comparison group 4/4) and laparoscopic necrosectomy with drainage (*n* = 7, the main group vs the comparison group 4/3) to resolve them. The median time for PCD insertion after the onset of abdominal pain was 15.7 [9,10,11,12,13,14,15,16,17,18,19,20,21] days in all patients. We would especially like to emphasize one more time that the drainages were inserted only when there was evidence of liquefaction on radiological imaging; solid debris and necrotic pancreatic tissue were not the intended targets. In 24 (16.2%) of the patients, the treatment was supplemented by laparoscopic necrosectomy and drainage, in 12 (8.1%), by video-assisted retroperitoneal debridement (VARD) and drainage, in 17 (11.5%), we performed operations using local laparotomy (after US or CT clarification/marking) with necrosectomy and drainage, and in 11 (7.4%) by decompressive VAC-laparostomy (Figure 6).

### 3.3. Features of Microbiological Studies

Pancreatic infection was documented in 92 (62.2%) out of the 148 patients. The fine needle aspirate taken from the pancreatic tissue was positive in 52 (56.5%) patients and the culture of pancreatic tissue obtained at surgery was positive in 40 (43.5%) patients. The sites of intrapancreatic and extrapancreatic infections found in 148 patients with acute pancreatitis are shown in Table 3.

Analyzing the results from 217 microbiological studies, microflora growth was absent in 22 cases (10.1%). Of the 195 strains, monomicrobial infections were isolated from 114 (58.5%) and 81 (41.5%) polymicrobial strains. The total number of strains identified was 314 (Table 4).

Gram-negative bacteria predominated in the general structure of pathogens with a share of 222 (70.7%) strains, of which 112 (50.5%) were represented by enterobacteria and 110 (49.5%) were NFGNB. The total proportion of gram-positive microorganisms was 92 strains (29.3%), among which enterococci predominated—46 strains (50%). It has been established that the main initiating etiological factor of the pancreatogenic infectious process is the autochthonous flora, the main part of which is gram-negative bacteria, primarily representatives of the Enterobacteriaceae. Gram-positive flora was detected in 54.3% of primary studies, with the predominant pathogens being enterococcal pathogens, in particular *Enterococcus faecalis* 28 (56%), and *Enterococcus faecium* 9 (18%). Microbial associations were found in 10 of 50 cases (20%) at the first culture of the wound discharge. In the treatment of pancreatic and extrapancreatic infections, 164 microbiological studies of discharge were taken (Table 3) for culture from the blood, urine, throat, intravenous cannula tip, urinary catheter tip, tracheal aspirate (in those on a ventilator), drain fluid (if instituted), and bile (if drained), and 240 cultures of microorganisms were isolated. An increase in microbial associations compared with the primary study was noted; a total of 74 polymicrobial associations were detected out of 240 cultures (30.8%). Among Gram-negative microorganisms, NFGNB began to predominate: *Pseudomonas aeruginosa* 65 (32.8%), *Acinetobacter* spp. 24 (12.1%). Along with them the following were present: *Klebsiella* spp. (18.2%), *E. coli* (12.1%), and *Proteus* spp. (8.1%). If at the beginning of treatment in the wound discharge, representatives of typical gram-positive intestinal flora (54.3%) prevailed, and among gram-positive microorganisms, pathogens were Enterococcus, in particular, *E. faecalis*—56%, *E. faecium*—18%, then an increase in the proportion of hospital was observed in dynamics, antibiotic-resistant strains of microorganisms. So, one week after the start of treatment, in 52.0% of cases in the main group and in 48.0% of cases in the comparison group, the wound discharge was contaminated with hospital antibiotic-resistant strains. Staphylococcus aureus began to predominate in the structure of gram-positive microorganisms after 7–14 days of treatment (33.3%), Staphylococcus epidermidis in 16.7%, and Staphylococcus saprophyticus in 14.3%, as well as in the associations of Enterococcus (24.1%). Differences in the spectrum of microflora and antibiotic resistance, depending on the localization of the purulent focus in pancreatic necrosis, were not revealed. Differences in the dynamics of the qualitative composition of the microflora, depending on the method of drainage of the purulent focus, were also not revealed. At the same time, differences in the clinical course of wounds contaminated with hospital microflora were revealed. The most common local infectious complication was the progression of inflammatory changes in soft tissues in the area of peripancreatic tissue and surgical access in 6 patients out of 95 (6.3%) in the main group and 22 out of 53 (41.5%) in the comparison group (χ2 = 15.900, *p* = 0.000).

Table 5 has given the sensitivity of organisms isolated from the pancreatic tissue and the extrapancreatic sites of the 92 patients.

### 3.4. Patient’s Outcomes

The principles of the ERAS concept were applied in the main group throughout the treatment of patients. The duration of treatment of patients after surgery in the intensive care unit was minimized (*p* = 0.004), and immediately after the compensation of vital functions, the patients were transferred to the surgical unit. The activation of patients began on the first day after the operation, and on the third day, they were already completely mobile. This became possible due to the active involvement of the physiotherapy service in the treatment process; all patients of the main group used the intake of as many clean liquids as possible on the first or second postoperative day. Statistically significant (χ2 = 19.697, *p* = 0.000) differences were found in the frequency of reduction in the number of complications in the main group of patients requiring conservative treatment or surgical treatment without general anaesthesia (Clavien-Dindo classes I–IIIa). In this group, postoperative complications have analyzed the frequency of acute venous thrombosis of the extremities, suppuration in the area of the postoperative wound, pleurisy, pneumonia, intra-abdominal fluid accumulation, etc. In case of the development of local infectious complications or progression of inflammatory changes in soft tissues in the area of peripancreatic tissue and surgical access, staged surgical treatment of the purulent focus and necrosectomy were performed. There were no statistically significant differences between the groups in terms of the total frequency of grade IIIb complications (fistulas of the small or large intestine, arrosive bleeding, secondary peritonitis) (χ2 = 1.283, *p* = 0.257).

**Figure 7:**
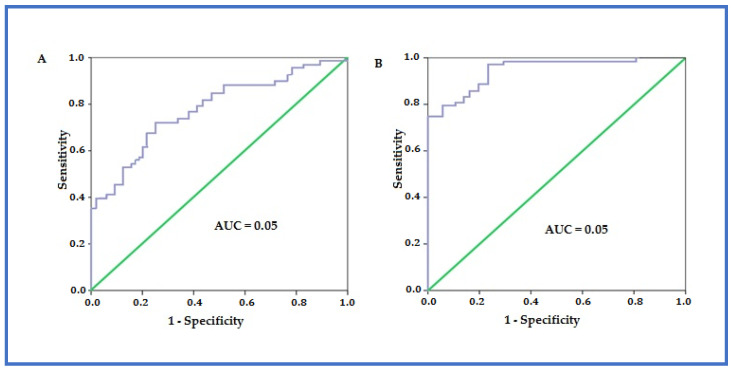
The area under of the receiver operating characteristic curve for the MOF before (**A**) and after (**B**) surgery.

The total proportion of patients with registered life-threatening complications of varying severity in the form of mono-organ or MOF (grades IVa and IVb) in the main group was 3.7% less than in the other group (χ2 = 2.628, *p* = 0.269): the impossibility of weaning from the ventilator  < 48 h (*n* = 17); septic shock (*n* = 8); myocardial infarction (*n* = 3); pulmonary embolism (*n* = 2). However, these differences were not significant. However, these differences were not significant. We also did not observe a significant effect of ERAS use on mortality in the main group compared to the comparison group (χ2 = 0.051, *p* = 0.081), and in regression analysis, we found a good dependence of postoperative mortality on the presence of MOF before surgery (AUC = 0.814, 0.95% CI 0.728–0.896) with a sensitivity of 81.2% and a specificity of 74.6%, and excellent dependence after surgery (AUC = 0.931, 0.95% CI 0.892–0.994) with a sensitivity of 89.2% and a specificity of 94.1% (Figure 7). With the help of active treatment using ERAS, it was possible to achieve a clinically and statistically significant effect on the duration of hospital treatment: in the main group, it was less than in the comparison group, on average, by 11 days (χ2 = 8.622, *p* = 0.003).

## 4. Discussion

It is known that the principles of surgical interventions for pancreatic necrosis were laid down by B. Moynihan in 1925 [18], and the main surgical methods for the control of ANP and sepsis over the past 40 years included: (1) “open technique” of treatment in the form of necrosectomy and open management of the source of infection [19]; (2) necrosectomy with planned relaparotomy of the source of infection [20]; (3) “closed technique” with necrosectomy, drainage and with continuous washing [21] or without it [22]. To this day, however, the principles of the treatment of necrotizing pancreatitis and the role of surgery remain controversial. During the 1990s, over 60% of ANP patients received open procedures [23].

Later, it was hypothesized that percutaneous drainage of infected pancreatic areas and fluid collections can have a positive therapeutic effect, and this was based on clinical observations that indicated that maximal removal of all necrotic tissue was not necessary for the successful management of patients with ANP. By draining infected fluid collections, the authors proved that the clinical condition of patients can improve after these interventions, and necrotic tissues can be successfully processed further by the patient’s immune system. In other words, the purpose of the drainage was to get rid of the infected fluid, not necrosis [24]. In the Netherlands, a group of researchers carried out a prospective, randomized and multicenter study called ‘A step-up approach or open necrosectomy for necrotizing pancreatitis’ [7]. After the diagnosis of necrotizing pancreatitis or infected necrosis of the pancreas, patients were randomly assigned to a group in which the tactics of a sequential ascending approach were used and a group in which patients underwent open surgical necrosectomy and drainage. In the main group of patients, the approach consisted of percutaneous or endoscopic drainage followed by minimally invasive VARD, if necessary, and remediation of the focus of infection until clinically and laboratory-confirmed improvement of the patient’s condition. About 35% of patients in the main group underwent only percutaneous drainage, and when using a step-up approach to the diagnosis and treatment of pancreatic infection, a decrease in the number of postoperative complications and mortality was achieved. M.C. van Baal et al. (2011) also cited the data of a meta-analysis that included 384 patients in whom percutaneous drainage of fluid accumulations in ANP was used as the main treatment method [25]. In this study, surgical necrosectomy was performed in 56% of patients and the overall mortality was 17%, but infected necrosis was confirmed in only 71% of patients.

Our study also found that 29.7% of 148 patients had focal, subtotal, and total infected necrosis, and 44.6% had local complications of ANP. In addition, our sample and the randomization of patients depended on only one factor: the use of the ERAS principle in accelerated approach tactics in the treatment of ANP. The duration of treatment of patients in the main group in the intensive care unit was minimized (*p* ≤ 0.004); statistically significant (9.5% vs 52.8%, *p* < 0.001) differences were found in the frequency of reduction in the number of complications of patients requiring conservative treatment or surgical treatment without general anaesthesia (Clavien-Dindo classes I-IIIa); there were no statistically significant differences of the total frequency of grade IIIb complications (*p* > 0.05); the total proportion of patients with registered life-threatening complications of varying severity in the form of MOF (grades IVa and IVb) in the main group was 3.7% less than in the other group (*p* = 0.269); the average duration of treatment for patients in the main group was 23 days, in the comparison group—34 days (*p* ≤ 0.003); however, we did not observe a significant effect of ERAS use on mortality in the main group compared to the comparison group (18.9% vs 22.6%, *p* > 0.05), which was more dependent on the presence of MOF before operation (AUC = 0.814) and after surgery (AUC = 0.931).

In the present study, we investigated pancreatic and extrapancreatic infections in patients with infected ANP. Pancreatic infections were observed in 92 patients and Gram-negative bacteria predominated in the general structure of pathogens with a share of 222 (70.7%) strains, of which 112 (50.5%) were represented by enterobacteria and 110 (49.5%) were NFGNB. The total proportion of gram-positive microorganisms was 92 strains (29.3%), among which enterococci predominated—46 strains (50%).

Limitations of the research. Our study has had several limitations. First, our data were based on patient medical records that were processed. Second, not all patients were accounted for in this study, but only those with a full set of biomarkers in their study profile. Thirdly, the patients with infected ANP were more valued, especially in the general population to better compare, and we had deliberately focused our study on these patients as these patients had been presenting the most difficult problem in the diagnosis and treatment. Certainly, in the ignored group there were patients who had died after surgery. As a result, bias in data selection could not be completely avoided and all results obtained require further verification in many more patients with ANP.

## 5. Conclusions

Differences in local resources, diagnostic and treatment options, institutional preferences, experience, and disease severity all contribute to the variability in the effectiveness of one or another approach to treating ANP. This study demonstrated the possibility of applying the principles of fast-track surgery in the complex treatment of ANP using a staged approach to treatment with a preference for minimally invasive methods, which can significantly reduce the number of Clavien-Dindo I-IIIa complications and the length of stay of patients in the hospital. MIP with ERAS is preferred in patients with limited necrotic accumulations and in patients who require intervention in the advanced stages of the disease when the necrosis is well liquefied. Lack of clinical improvement should always be an indication for open necrosectomy, which remains the preferred choice in situations such as acute pancreatitis complicated by abdominal compartment syndrome, colonic ischemia, and intestinal perforation.

## Figures and Tables

**Table 1:** Principles of the enhanced recovery pathway in ANP.

The Phases of Acute Pancreatitis	Enhanced Recovery Pathway (The Main Group)	Traditional Recovery Care (The Comparison Group)
The early phase (<1 week after onset)	Fluid management and analgesia; nutritional supplements; systemic support and indications for the intensive care unit admission	Fluid management and analgesia; nutritional supplements; systemic support and indications for the intensive care unit admission
	Routine endoscopic biliary drainage (plastic stent) for jaundice; laparoscopic evacuation of pancreatic ascites; the urinary catheter was removed (or replaced) for 3 days	Endoscopic retrograde cholangiography confirms the presence of stones; endoscopic papillosphincterotomy is performed; laparoscopic installation of drainage into the abdominal cavity is performed in the presence of pancreatic ascites; the urinary catheter removed on day 7–10 or replaced
The late phase (>1 week after onset)	Warm IV fluids, and air-warming device; analgesia pump; antibiotics; low-fat diet. Invasive interventions are shown for moderate and severe acute pancreatitis: puncture under the control of ultrasound, CT, or laparoscopy; drainage of fluid collections; necrosectomy for infected necrosis laparoscopically or using a laparotomy; VARD	Warm IV fluids, and air-warming device; analgesia pump; antibiotics; low-fat diet. Invasive interventions are shown for moderate and severe acute pancreatitis: puncture under the control of ultrasound, CT, or laparoscopy; drainage of fluid collections; necrosectomy for infected necrosis laparoscopically or using a laparotomy; VARD
Postoperative care in the intensive care unit	Treatment in the intensive care unit until the general condition the patient stabilizes	Treatment in the intensive care unit until the general condition the patient stabilizes
Postoperative care in the surgical department	Low-fat diet after transfer to the ward via nasojejunal tube; warm IV fluids; antibiotics; continued analgesia	Low-fat diet after transfer to the ward via nasojejunal tube; warm IV fluids; antibiotics; continued analgesia
Day 1	Start mobilization, breathing exercises; low-fat diet after transfer to the ward via nasojejunal tube; IV fluids; antibiotics; continued analgesia	Start mobilization, breathing exercises; low-fat diet after transfer to the ward via nasojejunal tube; IV fluids; antibiotics; continued analgesia
Day 2	Expand mobilization, breathing exercises	Expand mobilization, breathing exercises
Day 3	Expand mobilization, breathing exercises; removal of the nasogastric tube if discharge < 200 mL; taking clear liquids by mouth; the urinary catheter was removed; continue to mobilize at least four times a day; taking clear liquids by mouth; the urinary catheter was removed; continue to mobilize at least four times a day	Continue to mobilize at least four times a day; low-fat diet via nasojejunal tube
Day 4	Oral ingestion of conventional diet, an extension of mobilization, and assessment of severity criteria	Low-fat diet via nasojejunal tube, and assessment of severity criteria
Day 5–10	Stop elastomeric pump; removal of drainage tubes if no pancreatic fistula and <200 mL	Removal of the nasogastric tube; the urinary catheter was removed; stop elastomeric pump
Day 10–14	No fever for more than 48 h, normal procalcitonin levels, consider transferring to follow-up with a physician in the community: the patient is able to take solid food; has a normal stool; has adequate mobilization	No fever for more than 48 h, normal procalcitonin levels: removal of drainage tubes in the absence of pancreatic fistula and <200 mL; change of drainage tubes to a smaller diameter with a discharge of >200 mL; continue or change of antibiotics if a fever continue 200 mL; change of drainage tubes to a smaller diameter with a discharge of >200 mL

**Note:** If the patient’s condition worsens, all therapeutic measures are resumed; perform repeated operations according to indications; change antibiotics according to the results of bacteriological examination. The day of surgery in any repeated laparoscopic or laparotomy was considered zero.

**Table 2:** Demographic, clinical, and laboratory characteristics of patients with ANP.

Indicators	Comparison Group (*n* = 53)	Main Group (*n* = 95)	*p*
Age (years), median (IQR)	53 (29–69)	57 (36–70)	0.062
BMI kg/m^2^, median (IQR)	26 (21–32)	27 (22–33)	0.126
M/F, *n* (%)	32/21 (60.4%/39.6%)	56/39 (58.9%/41.1%)	0.986
Etiology of AP, *n* (%): Alcohol Cholelithiasis Other	34 (64.1%) 17 (32.1%) 2 (3.8%)	57 (60%) 35 (36.8%) 3 (3.2%)	1.000
Primarily operated in other hospitals, *n* (%)	9 (17%)	15 (15.8%)	0.945
BISAP score, median (IQR)	4 (2–5)	4 (2–5)	0.664
SOFA score, median (IQR)	8 (6–12)	8 (5–12)	0.399
APACHE II score, median (IQR)	12 (6–22)	14 (7–24)	0.432
The prevalence of pancreatic necrosis, *n* (%): <30% 30–50% >50%	10 (18.9%) 31 (58.5%) 12 (22.6%)	21 (22.1%) 55 (57.9%) 19 (20%)	1.000
Fever, ≥38.5 °C, *n* (%)	19 (35.8%)	35 (36.8%)	0.933
WBC count, ≥12.2 × 10^9^/L, *n* (%)	34 (64.2%)	63% (66.3%)	0.988
Blood lactate ≥2 mmol/L, *n* (%)	24 (45.3%)	47 (49.5%)	0.889
PCT level (ng/mL), *n* (%): <2, *n* (%) 2–10, *n* (%) ≥10, *n* (%):	19 (35.8%): 9 (47.4%) 9 (47.4%) 1 (5.2%)	34 (35.8%): 18 (52.9%) 14 (41.2%) 2 (5.9%)	1.000
Transient organ failure, *n* (%)	32 (60.4%)	56 (58.9%)	0.956
Permanent organ failure, *n* (%)	21 (39.6%)	39 (41.1%)	0.961
Sterile ANP, *n* (%)	15 (28.3%)	23 (24.2%)	0.819
Infected ANP, *n* (%)	12 (22.6%)	32 (33.8%)	0.385
Local complications, *n* (%)/(sterile/infected) Including: ANFC (%)/(sterile/infected) APPFC (%)/(sterile/infected) WON (%)/(sterile/infected) PS (%)/(sterile/infected)	26 (49.1%)/(6/20) 12 (46.2%)/(3/9) 5 (19.2%)/(2/3) 7 (26.9%)/(0/7) 2 (7.7%)/(1/1)	40 (42.1%)/(12/28) 19 (47.5%)/(7/12) 8 (20%)/(2/6) 8 (20%)/(0/8) 5 (12.5%)/(3/2)	0.953
Ventilation support, *n* (%)	24 (45.3%)	39 (41.5%)	0.874
Inotropic support, *n* (%)	23 (43.9%)	35 (36.8%)	0.724
Artificial kidney, *n* (%)	6 (11.3%)	9 (9.5%)	0.968
Stay in ICU, median (IQR)	11 (1–18)	3 (1–8)	0.004
Postoperative complications: Clavien-Dindo I, *n* (%) Clavien-Dindo II, *n* (%) Clavien-Dindo IIIa, *n* (%) Clavien-Dindo IIIb, *n* (%) Clavien-Dindo IVa, *n* (%) Clavien-Dindo IVb, *n* (%) Clavien-Dindo V, *n* (%)	10 (18.9%) 12 (22.6%) 6 (11.3%) 2 (3.8%) 3 (5.7%) 9 (17%) 12 (22.6%)	2 (2.1%) 4 (4.2%) 3 (3.2%) 0 1 (1.1%) 17 (18%) 18 (18.9%)	0.003
Duration of treatment in a hospital, days, median (IQR)	34 (12–44)	23 (10–29)	0.003

**Table 3:** Sites of intrapancreatic and extrapancreatic infections found in 148 patients with acute pancreatitis.

Site	Number of Causes (%)
Pancreatic & Peripancreatic	92 (62.2%)
Blood culture	24 (16.2%)
Ascitic fluid culture	15 (10.1%)
Bile fluid culture	3 (2.02%)
Pleural fluid culture	13 (8.8%)
Urine catheter culture	19 (12.8%)
Intravenous catheter culture	17 (11.5%)
Drain fluid culture	76 (51.4%)
Tracheal aspirate culture	15 (11.1%)
Wound culture	40 (24.3%)

**Table 4:** Microbiology of cases of infected pancreatic necrosis.

Type of Microflora	Total	%	Number of Strains of Microorganisms			
			Initial Identification of Bacteria		In the Dynamics of Treatment (after 1–2 Weeks)	
			Total	%	Total	%
Gram-negative flora	222	70.7	24	10.8	198	89.2
*E. coli*	35	15.8	11	45.8	24	12.1
*Proteus spp.*	18	8.1	2	8.3	16	8.1
*Klebsiella spp.*	46	20.7	10	41.7	36	18.2
*Other enterobacteriaceae*	13	5.9	1	4.2	12	6.1
*Pseudomonas aeruginosa*	65	29.3	0	0	65	32.8
*Acinetobacter spp.*	24	10.8	0	0	24	12.1
*Other NFGNBs* *	12	5.4	0	0	12	6.1
*Other gram-negative bacteria*	9	4.1	0	0	9	4.5
Gram-positive flora	92	29.3	50	54.3	42	45.7
*Staphylococcus aureus*	18	19.6	4	8	14	33.3
*Staphylococcus epidermidis*	9	9.8	2	4	7	16.7
*Staphylococcus saprophyticus*	7	7.6	1	2	6	14.3
*Enterococcus faecalis*	34	37	28	56	6	14.3
*Enterococcus faecium*	12	13	9	18	3	7.1
*Other gram-positive bacteria*	12	13	6	12	6	14.3
Total:	314		74 (23.6%)		240 (76.4%)	

* NFGNB, nonfermenting gram-negative bacilli.

**Table 5:** Characteristics of sensitivity to antibiotics of cultivated pathogens.

Microorganism	Antibiotic Sensitivity
*E. coli* (*n* = 35)	Amikacin (26; 74.3%), Imipenem (29; 82.9%), Meropenem (30; 85.7%), Pipercillin/tazobactum (17; 48.6%), Netilmicin (11; 31.4%), Cefuroxime (23; 65.7%) Ceftriaxone (25; 71.4%%), Cefoperazone (3; 8.6%), Cefoperazone/sulbactam (16; 45.7%), Ciprofloxacin (26; 74.3%)
*Proteus* spp. (*n* = 18)	Colistin (16; 88.9%), Ceftazidime/avibactam (18; 100.0%), Tigecycline (18; 100.0%), Cefoperazone/sulbactam (9; 50.0%), Ciprofloxacin (5; 27.8%), Moxifloxacin (9; 50.0%); Amikacin (8; 44.4%)
*Klebsiella* spp. (*n* = 46)	Imipenem (32; 69.6%), Meropenem (41; 89.1%), Pipercillin/tazobactum (28; 50.0%), Ciprofloxacin (22; 47.8%), Cefotaxime (11; 23.9%), Ceftazidime (10; 21.7%)
*Pseudomonas aeruginosa* (*n* = 65)	Imipenem (23; 35.4%), Meropenem (26; 40.0%), Ceftazidime/avibactam (65; 100%), Pipercillin/tazobactum (29; 44.6%), Netilmicin (18; 27.7%), Cefepime (34; 52.3%)
*Acinetobacter* spp. (*n* = 24)	Imipenem (10; 41.7%), Meropenem (14; 58.3%), Pipercillin/tazobactum (9; 37.5%), Amikacin (7; 29.2%), Netilmicin (3; 12.5%), Ciprofloxacin (4; 16.7%), Cefoperazone/sulbactam (13; 54.2%), Cefotaxime (2; 8.3%), Ceftazidime (2; 8.3%)
*Staphylococcus aureus* (*n* = 18)	Amikacin (4; 22.2%), Netilmicin (3; 16.7%), Ciprofloxacin (8; 44.4%), Vancomycin (14; 77.8%), Cefotaxime (2; 11.1%), Clindamycin (12; 66.7%), Linezolid (16; 88.9%), Tigecycline (17; 94.4%)
*Staphylococcus epidermidis* (*n* = 9)	Imipenem (4; 44.4%), Meropenem (5; 55.6%), Amikacin (1; 11.1%), Netilmicin (3; 33.3%), Ciprofloxacin (2; 22.2%), Vancomycin (4; 44.4%), Cefotaxime (2; 22.2%), Clindamycin (6; 66.7%), Linezolid (9; 100.0%); Teicoplanin (5; 55.6%); Tigecycline (9; 100.0%)
*Staphylococcus saprophyticus* (*n* = 7)	Ampicillin/sulbactam (6; 85.7%), Amoxicillin/clavulanate (6; 85.7%), Netilmicin (5; 71.4%), Ciprofloxacin (1; 14.3%), Vancomycin (7; 100.0%), Cefotaxime (2; 28.6%), Clindamycin (2; 28.6%), Tigecycline (7; 100.0%); Pipercillin/tazobactum (7; 100.0%)
*Enterococcus faecalis* (*n* = 34)	Vancomycin (16; 47.1%), Pipercillin/tazobactum (12; 35.3%), Tigecycline (34; 100.0%); Linezolid (34; 100.0%)
Enterococcus faecium (*n* = 12)	Vancomycin (5; 41.7%), Pipercillin/tazobactum (3; 25.0%), Tigecycline (12; 100.0%); Linezolid (12; 100.0%)

## Data Availability

All data associated with this study are present in the paper.

## Abbreviations

ACS	abdominal compartment syndrome
ANFC	acute necrotic fluid collections
ANP	acute necrotizing pancreatitis
AP	acute pancreatitis
APACHE II	Acute Physiology and Chronic Health Evaluation II score
APP	abdominal perfusion pressure.
APPFC	acute pancreatic/peripancreatic fluid collections
AUC	Area Under the Curve
CT	computed tomography
ERAS	Enhanced Recovery After Surgery
F	female
IPN	infected pancreatic necrosis
LD	laparoscopic drainage
M	male
MIP	minimally invasive procedures
MOF	multiple organ failure
NFGNB	nonfermenting gram-negative bacilli
PCD	percutaneous catheter drainage
PCT	procalcitonin
PS	postnecrotic pseudocyst
ROC	receiver operating characteristic
SOFA	Sequential Organ Failure Assessment score
VARD	video-assisted retroperitoneal debridement
WBC	white blood cells
WON	walled-off necrosis
